# Severe combined imunodeficiency caused by defect of the common gamma chain of the interleukin 2 receptor

**DOI:** 10.1186/1939-4551-8-S1-A133

**Published:** 2015-04-08

**Authors:** Luanda De Alleluia, Flavia Anisio, Liziane Nunes, Paula Lauria, Sandra Bastos, Abelardo Neto, Anna Luiza Paola Martins, Livia Lucas Lima, Celso Ungier

**Affiliations:** 1 Instituto Nacional De Saude Da Mulher, Da Criança e Do Adolescente Fernandes Figueira - IFF/ Fiocruz, Brazil

## Background

Severe combined immunodeficiency (SCID) is the most serious form of a group of diseases characterized by an abnormality in the development and / or function of T cells and may be associated with defects in B cells and Natural Killer cells.

## Methods

Case report of a 2 years old male diagnosed with Severe Combined Immunodeficiency (SCID) at 5 months of age investigated to define the molecular basis of the disease due the untimely death of two siblings.

## Results

The mutation detected was a defect of the common gamma chain of the interleukin 2 receptor (IL2Rγ). Even though genetic counseling advised otherwise the patient's mother got pregnant during follow-up and as no compatible donor was found we chose to wait birth and verify compatibility. Genetic evaluation of the newborn revealed the absence of the IL2Rγ gene defect in blood cord and a matching HLA. Cord stem cell transplantation was scheduled afterwards.

## Conclusions

The patient's mutation is the most common variant (IL2Rγ gene defect) in the X-linked expressed pattern of the XL T-B+NK-phenotype which corresponds to about 45% of severe combined immunodeficiency according to the literature.

## Consent

Written informed consent was obtained from the patient for publication of this abstract and any accompanying images. A copy of the written consent is available for review by the Editor of this journal.

